# A blood‐based multi‐modal assay for diagnosing Alzheimer's disease

**DOI:** 10.1002/alz.089477

**Published:** 2025-01-09

**Authors:** Souvik Modi, Hernando M. Vergara, Shareefa Thekkan, Leonidas Chouliaras, Maura Malpetti, Peter Swann, James B Rowe, John T O'Brien

**Affiliations:** ^1^ Esya Labs, London UK; ^2^ ESYA labs, London, London UK; ^3^ University of Cambridge, Cambridge UK; ^4^ Cambridge University Hospitals NHS Foundation Trust, Cambridge UK; ^5^ Department of Clinical Neurosciences and Cambridge University Hospitals NHS Trust and Medical Research Council Cognition and Brain Sciences Unit, University of Cambridge, Cambridge UK; ^6^ Department of Psychiatry, University of Cambridge, Cambridge UK; ^7^ Cambridgeshire and Peterborough NHS Foundation Trust, Cambridge UK

## Abstract

**Background:**

Combinations of blood‐based biomarkers have been used to detect Alzheimer’s disease (AD). While these markers provide information about neuropathology, they fail to integrate the cellular dysfunction, such as disease‐associated defects in lysosomal ion homeostasis. To understand cellular dysfunction in AD and its relation to the pathophysiology of the disease, we developed a multi‐modal biomarker diagnostic platform that incorporates lysosomal ionic pH and Ca^2+^ and plasma levels of Amyloid beta_1‐42_ (Aβ_1‐42_), Amyloid beta_1‐40_ (Aβ_1‐40_), phosphorylated Tau181 (pTau181), Neurofilament light (NfL) and Glial fibrillary acidic protein (GFAP).

**Method:**

This multi‐modal blood‐based platform incorporates the concentrations of H^+^ and Ca^2+^ ions with single lysosome resolution in monocytes measured using DNA nanodevices from 33 participants (20 Controls and 13 AD patients). It also includes a panel of pathological biomarkers, including Aβ_1‐40_, Aβ_1‐42_, pTau181, NfL, and GFAP, which are measured using the Simoa platform from plasma across seven different cohorts totalling 323 participants (146 Controls, 92 AD patients, 34 amyloid negative and 51 amyloid positive mild cognitive impairment).

**Result:**

The minimalistic panel of GFAP and pTau181/Aβ_1‐42_ ratio performs significantly better (area under the curve [AUC] = 85%) in predicting Amyloid PET status than either NfL, GFAP, Aβ_1‐42_/Aβ_1‐40_ ratio or pTau181 alone, with AUCs ranging from 68‐77%. The performance of these individual biomarkers or their combinations were further enhanced when lysosomal ionic information was incorporated, differentiating AD subjects from healthy individuals with an accuracy of 88% (Sensitivity 85%; Specificity 95%).

**Conclusion:**

The multi‐modal blood platform described here is highly accurate, specific, and sensitive in evaluating AD, representing a promising avenue to explore the connections between cellular biology and the pathological hallmarks of AD, and the diagnosis of this disease.

